# Supplementary material for: Mesoscopic landscape of cortical functions revealed by through-skull wide-field optical imaging in marmoset monkeys
Source: Nat Commun. 2022 Apr 26;13:2238. doi: 10.1038/s41467-022-29864-7 (PMC9042927; doi:10.1038/s41467-022-29864-7)
Supplement: Supplementary file 1 — Supplementary Information [file 41467_2022_29864_MOESM1_ESM.pdf]

Supplementary Information for

Mesoscopic landscape of cortical functions revealed by through-skull wide-field optical imaging in marmoset monkeys

Xindong Song*, Yueqi Guo, Hongbo Li, Chenggang Chen, Jong Hoon Lee, Yang Zhang, Zachary Schmidt, and Xiaoqin Wang*

*Correspondence to: songxindong@jhmi.edu, xiaoqin.wang@jhu.edu.

This PDF file includes:

Supplementary Table 1. Summary of subjects used towards landscape mapping.

Supplementary Fig. 1. The effect of wavelength on identifying cortical modalities.

Supplementary Fig. 2. The effect of wavelength on identifying tonotopy in the auditory cortex.

Supplementary Fig. 3. The effect of simultaneous paradigm on modality parcellation.

Supplementary Fig. 4. Modality parcellation in all tested subjects.

Supplementary Fig. 5. Tonotopy mapping in all tested subjects towards landscape mapping.

Supplementary Fig. 6. Retinotopy and motion- sensitivity mapping in all tested subjects.

Supplementary Fig. 7. Face patch mapping stimuli.

Supplementary Fig. 8. Through-skull somatotopy mapping.

Supplementary Fig. 9. Face patch mapping in all tested subjects and their cortical functional landscapes.

Supplementary Fig. 10. Registrations of the PD face patch to the retinotopic and motion sensitivity maps.

Supplementary Fig. 11. Quantification of individual variation in the functional landscape.

Subject ID	M126D	M15E	M8E	M7E	M44D	M117B
Gender	female	male	male	male	male	male
Imaging side	left	left	right	left	right	right
Chamber bottom material	orthodontic resin	C&B metabond	orthodontic resin	orthodontic resin	orthodontic resin	C&B metabond
Age at the head cap implantation (month)	36	43	45	44	38	54
Age at the earliest imaging acquisition (month)	47	44	46	46	54	80
Age at the latest imaging acquisition (month)	52	48	48	46	55	81
Experiment participation: modality parcellation	Y	Y	Y	Y	Y	Y
Experiment participation: somatotopy	Y	Y	Y	N/A[1]	Y	N/A[2]
Experiment participation: tonotopy	Y	Y	Y	Y	Y	N/A[3]
Experiment participation: retinotopy	Y	Y	N/A[4]	Y	Y	Y
Experiment participation: face patch	Y	Y	N/A[4]	N/A[1]	N/A[5]	Y
Availability comments	[1] The subject was not available anymore when these experiments were designed.<br>[2] The parcellation experiment showed the recording chamber of this subject included very little somatosensory cortex for further investigations.<br>[3] The skull over part of the auditory cortex in this subject was thinned in an earlier pilot experiment. Thus tonotopy mapping through the intact unthinned skull could not be performed anymore in this subject<br>[4] Pursuit eye movement behavior in this subject was observed abnormal comparing to that in other subjects (confirmed by eye tracking). Thus the subject was excluded from further visual experiments<br>[5] The posteroventral part of the chamber was not big enough to include the full region of interest in this subject, based on the results of the retinotopy mapping experiment. Thus further face patch searching was not performed					

Supplementary Table. 1. Summary of subjects used towards landscape mapping.
A breakdown summary of all six tested subjects towards landscape mapping in the current study, including one female, five males; three left hemispheres, three right hemispheres; four with chamber bottom covered with orthodontic resin, two with chamber bottom covered with C&B Metabond. Subjects' ages ranged from 36 to 54 months old at the time of head cap implantation, and from 44 to 81 months old during imaging acquisition. Out of 30 possible experiments (5 experiments × 6 subjects), 7 were not conducted due to the availability issues commented on in the table.

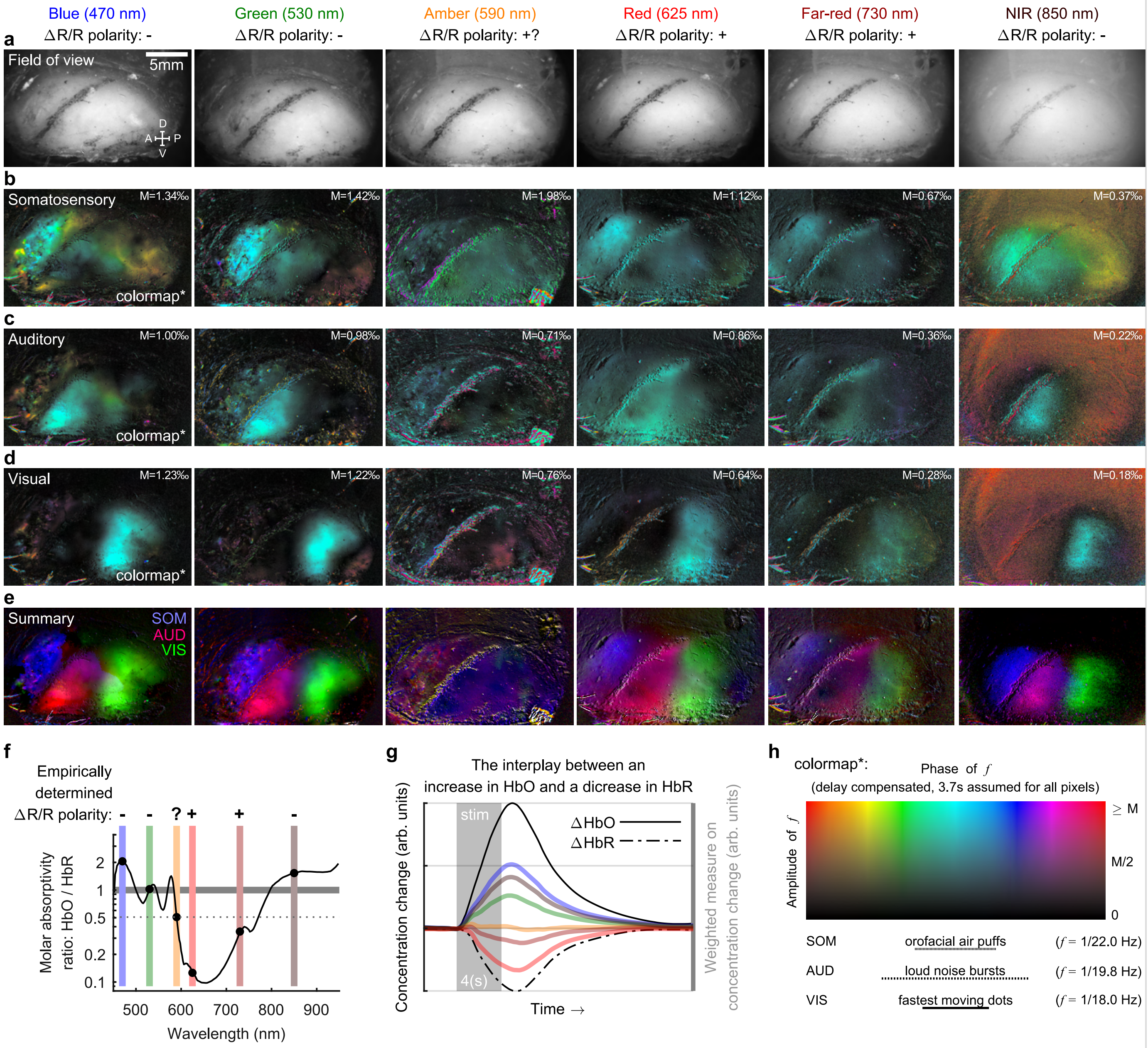

Supplementary Fig. 1. The effect of wavelength on identifying cortical modalities

a. The images of the recording chamber, taken with six different illumination wavelengths in subject M126D. The color, the wavelength, and the summarized signal polarity of each illumination light are indicated at the top of each column. A: anterior; P: posterior; D: dorsal; V: ventral. **b-d**. The somatosensory (**b**), auditory (**c**), and visual (**d**) response maps, acquired with each wavelength following the Fig. 2 experiment, and with ΔR/R signal polarity compensated. Response phase and amplitude are visualized separately as color and intensity in these maps, according to the 2D colormap in (**h**). The shorter wavelengths (blue and green) had superior response amplitudes over other wavelengths for all three modalities. **e**. A summary map for each wavelength, based on the results in (**b-d**), with SOM, AUD, and VIS in the blue, red, and green channels, respectively. The response patterns of each modality are generally consistent among all wavelengths and agree with the atlas (Fig. 1b). The maps acquired with the amber light show ambiguious response patterns regardless of which polarity (+ or -) was assumed. The auditory response patterns measured with blue, green, and NIR lights are generally similar but are different from the patterns measured with red and far-red lights (see also the "determining the experimental wavelength" and "intrinsic signal polarity" sections in Methods). **f**. Observed signal polarity and HbO/HbR molar absorptivity ratio. The empirically determined ΔR/R polarities of tested wavelengths (vertical color stripes) are labeled at the top (see also Fig. 1i). The solid thick (dashed thin) horizontal line represents a molar absorptivity ratio of 1 (0.5) for reference. Observed signal polarity is correlated with the molar absorptivity ratio in a way that any wavelength with a ratio higher than 0.5 (blue, green, and NIR) shows a negative signal polarity (-); any wavelength with a ratio lower than 0.5 (red, far-red) shows a positive signal polarity (+); and the amber light with a ratio of ~0.5 shows ambiguity in signal polarity. **g**. A hemodynamic model for explaining the wavelength effect on signal polarity. The major hemodynamic response following a stimulus was suggested[88] to consist of an HbO increase (the black solid curve) and an HbR decrease (the black dash curve). Assuming the HbO increase is twice in amplitude as the HbR decrease, the wavelengths with higher ratios (blue, green, and NIR) would pick up the HbO increase more over the HbR decrease and are thus more absorbed during the hemodynamic response (negative ΔR/R), whereas the wavelengths with lower ratios (red and far-red) would pick up the HbR decrease more over the HbO increase and are thus less absorbed during the hemodynamic response (positive ΔR/R). The amber light may pick up these opposite changes in a more balanced way and is thus ambiguous in signal polarity. **h**. The 2D colormap for the response maps shown in (**b-d**).

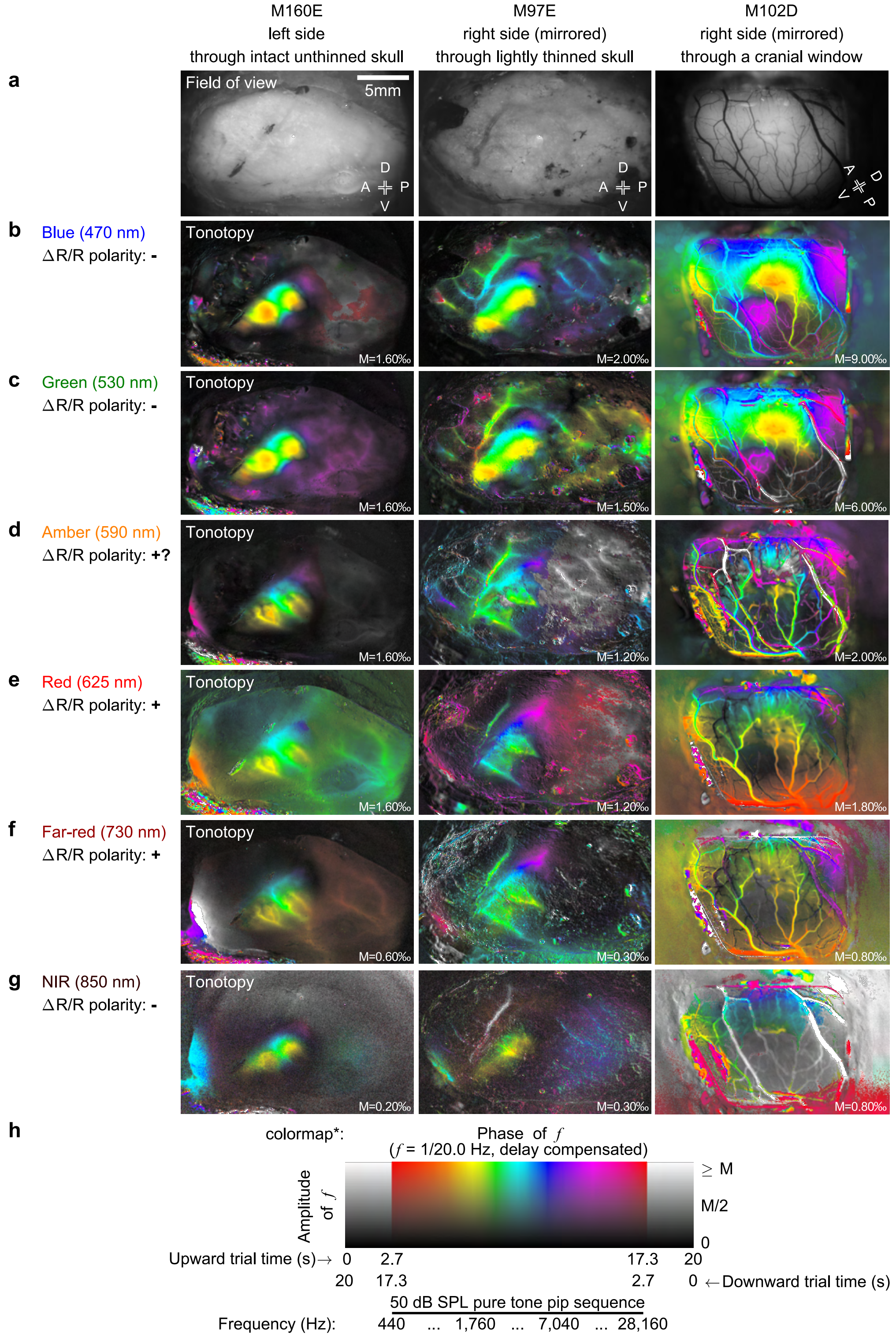

Supplementary Fig. 2. The effect of wavelength on identifying tonotopy in the auditory cortex.
a. The field of view in each subject, taken with the green light. The subject ID, the hemisphere side, and the type of imaging preparation are indicated at the top of each column. Imaging preparation varies in these subjects: through the un-thinned intact skull in subject M160E, through the lightly thinned skull in subject M97E, and through an implanted cranial window without the skull in subject M102D. The right hemisphere view is mirrored to match the view of the left hemisphere for display purposes. A: anterior; P: posterior; D: dorsal; V: ventral. **b-g**. The tonotopy mapping results with the blue (**b**), green (**c**), amber (**d**), red (**e**), far-red (**f**), and near-infrared (**g**) illumination lights. Each map was acquired following the same experiments presented in Fig. 3. Each row shows experimental results acquired with the same illumination light (wavelength), as indicated at the left of each row. All tonotopic maps are compensated for assumed ΔR/R signal polarity (also summarized at the left of each row). The hemodynamic delay is estimated and compensated for each pixel individually in these maps. Response phase and amplitude are visualized separately as color and intensity in these maps, according to the 2D colormap in (**h**). For each response map, the upper display limit of response amplitude “M” is listed at the lower right. Similar as in Supplementary Fig. 1, the shorter wavelengths (blue and green) had superior response amplitudes over the other wavelengths in all three subjects. The amber, red, and far-red wavelengths produce maps that are relatively more biased by the vasculature structures. **h**. The 2D colormap for the response maps shown in (**b-g**).

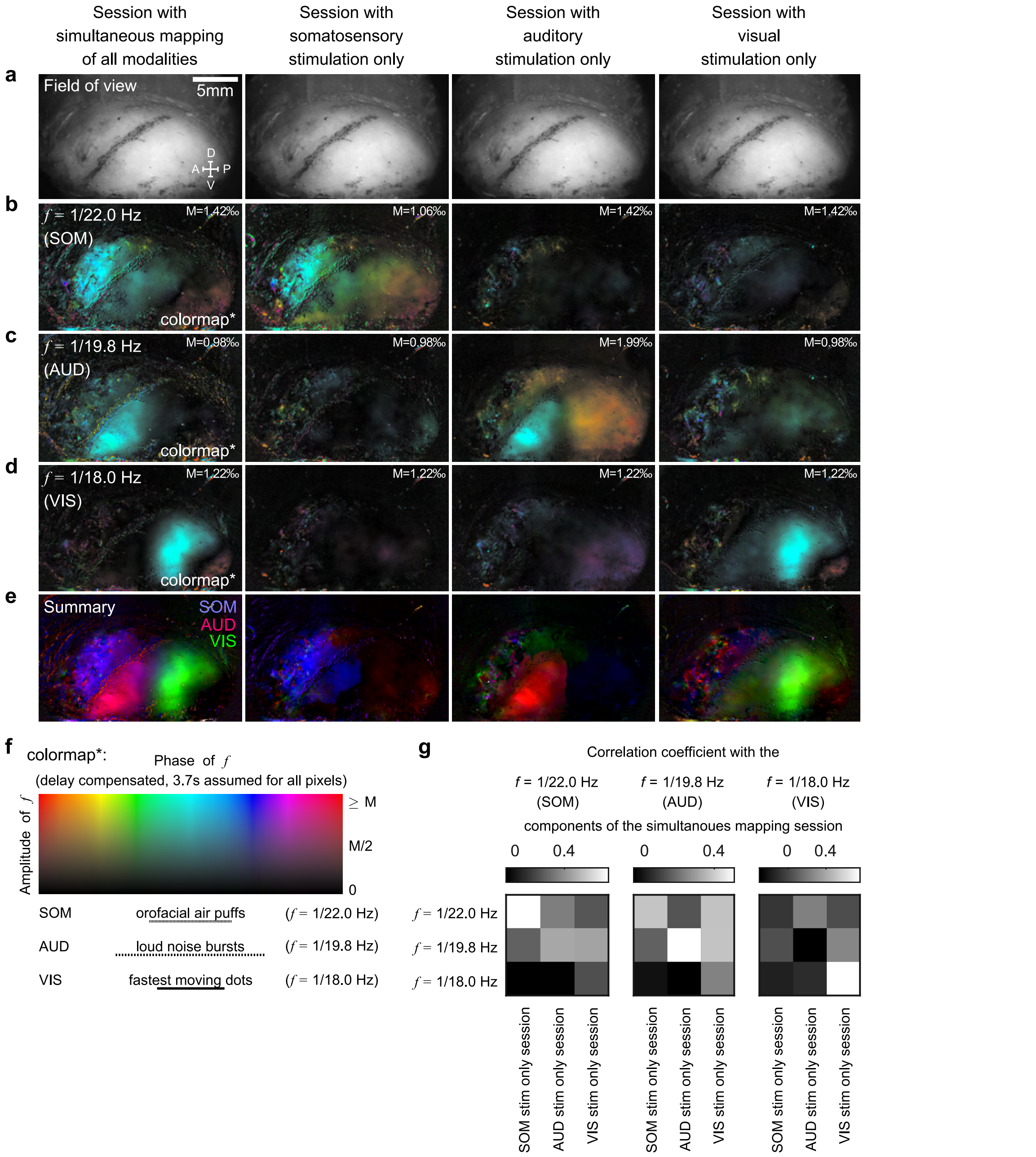

Supplementary Table. 3. The effect of simultaneous paradigm on modality parcellation.

a. The field of view in each session. Four sessions are shown in four columns, with each session's stimulation specified at the top of the corresponding column (all in subject M126D). The first session is the same as the experiment presented in Fig. 2 that parcellated somatosensory, auditory, and visual cortices simultaneously. The rest three sessions each had only one of the three stimulation modalities from the Fig. 2 experiment, to test each sensory modality individually. A: anterior; P: posterior; D: dorsal; V: ventral. **b-d**. The response maps of the f=1/22.0 Hz (**b**), f=1/19.8 Hz (**c**), and f=1/18.0 Hz (**d**) components of each session. These components correspond to the designed repetition cycles of the somatosensory, auditory, and visual stimuli, respectively. Response phase and amplitude are visualized separately as color and intensity in these maps, according to the 2D colormap in (**f**). For each response map, the upper display limit of response amplitude "M" is listed at the top right. The individually mapped results show clear activations when the corresponding stimulation was presented and little activation when the stimulation was missing. **e.** A summary map for each session. The map is based on the modality-specific results in (**b-d**), with SOM in the blue color channel, AUD in the red color channel, and VIS in the green color channel. The individually mapped sessions only show clear activations in the color channel corresponding to the presented stimulation modalities. **f.** The 2D colormap for the response maps shown in (**b-d**). **g.** The correlation coefficients between the components of the simultaneously mapped session (1st column in **b-d**) and the components of individually mapped sessions (2nd to 4th columns in **b-d**). For each component of the simultaneously mapped session, the correlation coefficient reaches its peak with the component of the individually mapped session at which the corresponding stimulation was presented. These results suggest the individually mapped activation patterns are very similar to their simultaneously mapped counterparts, thus verifying the patterns we have measured in Fig. 2 were largely unaffected by the simultaneous through-skull mapping paradigm.

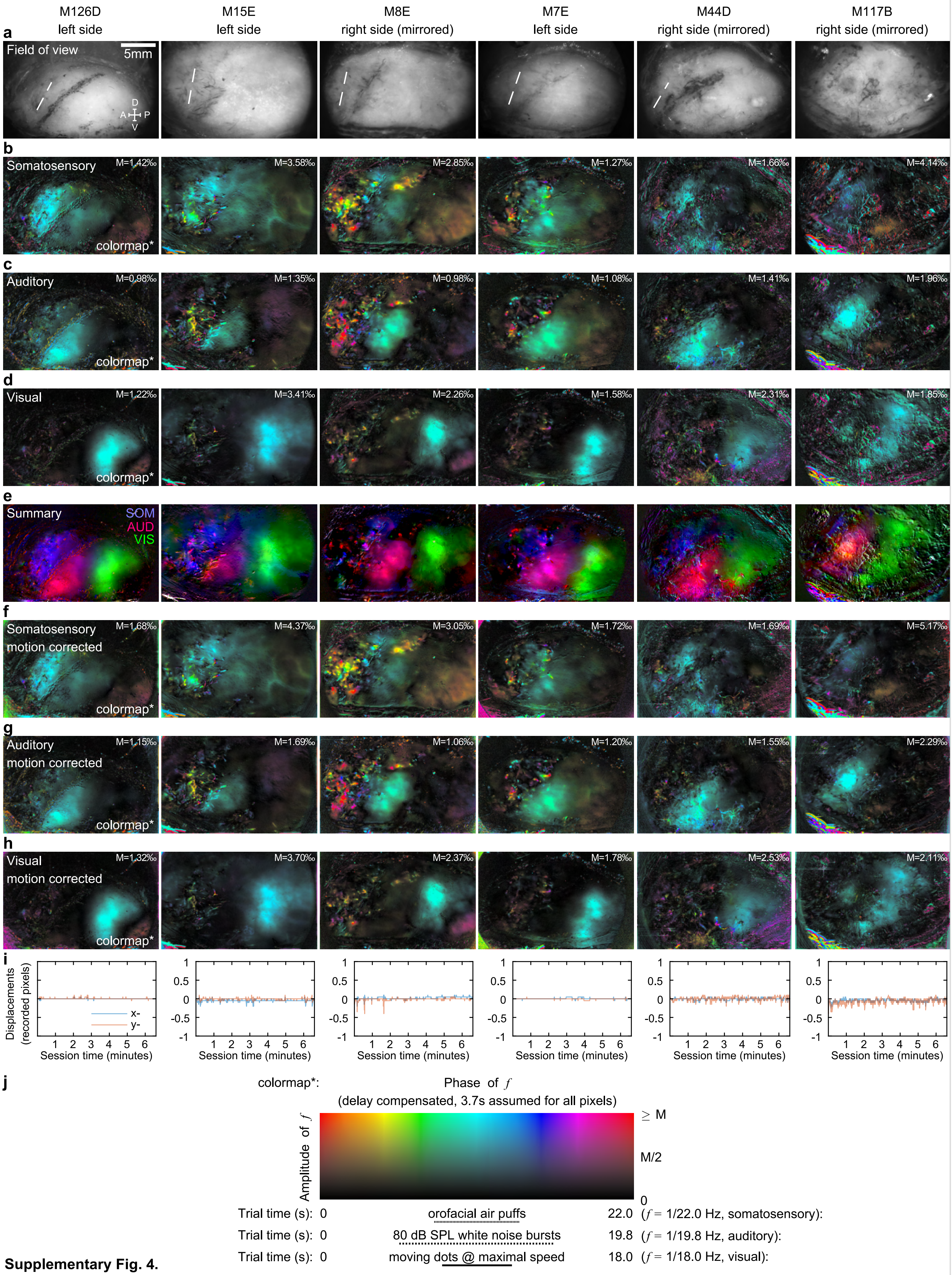

Supplementary Fig. 4.

Supplementary Fig. 4. Modality parcellation in all tested subjects

a. The field of view in each subject. Six tested subjects are shown here in six columns, with each subject's ID and the imaged hemisphere indicated at the top of the corresponding column. The right hemisphere view is mirrored to match the view of the left hemisphere for display purposes. Dashed line: the putative location of the coronal suture. A: anterior; P: posterior; D: dorsal; V: ventral. **b**-**d**. The somatosensory (**b**), auditory (**c**), and visual (**d**) response maps from each subject. Maps were acquired following the modality parcellation experiment presented in Fig. 2. Each column shows experimental results acquired in an individual subject, as indicated at the top of each column above (**a**). A fixed hemodynamic delay value of 3.7 seconds is assumed and compensated for all pixels in these maps. Response phase and amplitude are visualized separately as color and intensity in these maps, according to the 2D colormap in (**j**). For each response map, the upper display limit of response amplitude "M" is listed at the top right. **e**. A summary map for each subject. The map is based on the modality-specific results in (**b**-**d**), with SOM in the blue color channel, AUD in the red color channel, and VIS in the green color channel. These response patterns are generally consistent with the atlas (Fig. 1b). Subject M117B had a chamber that barely covered any part of the somatosensory cortex and had part of the skull (~3 mm × 4 mm) over the auditory cortex thinned in an earlier pilot experiment. Compared to the other subjects, M117B had a higher response amplitude in this part of the auditory cortex. **f**-**h**. The motion-corrected counterparts of the response maps in (**b**-**d**). **i**. The amounts of x- and y- displacements in individual recorded frames (480×300@80fps) estimated by the motion correction. These displacements are at sub-pixel scales in each of the subjects (size of recorded pixel: 46.9 um). **j**. The 2D colormap for the response maps shown in (**b**-**d** and **f**-**h**).

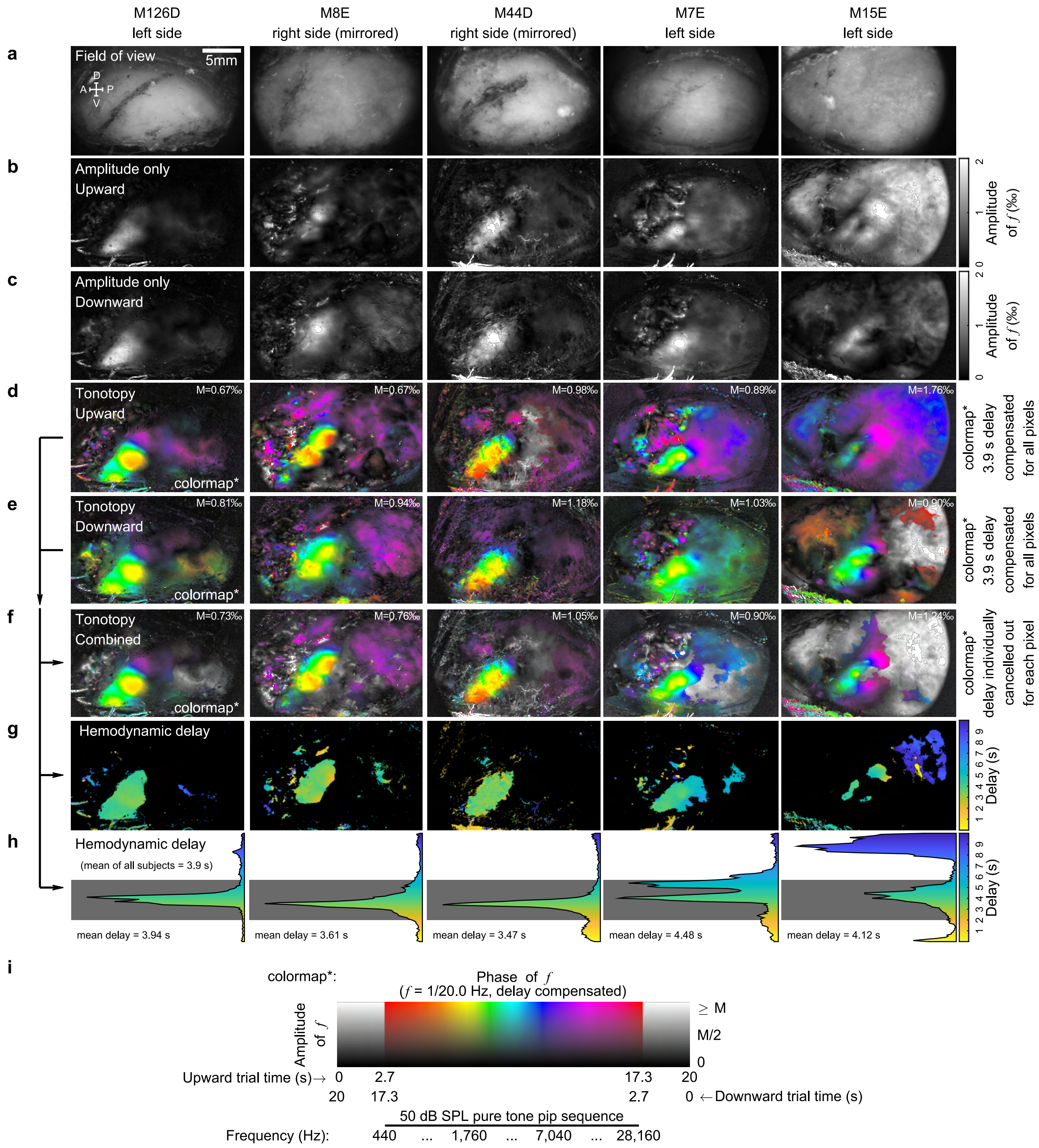

Supplementary Fig. 5. Tonotopy mapping in all tested subjects towards landscape mapping
a. The field of view in each subject. Column title: subject's ID and imaged hemisphere side. A: anterior; P: posterior; D: dorsal; V: ventral. **b-c**. Amplitude-only response maps of the session with an upward pure-tone pip sequence (**b**) or a downward pure-tone pip sequence (**c**), following the tonotopy mapping experiments presented in Fig. 3. The region that is consistent with the location of the auditory cortex (Fig. 1b) exhibits the highest response amplitude in most of these maps. The two sessions (**b-c**) performed in the same subject generally produce very similar results. **d-e**. Tonotopic maps derived from the session with an upward pure-tone pip sequence (**d**) or with a downward pure-tone pip sequence (**e**). The tuning phase and amplitude are visualized separately as color and intensity in these maps, according to the 2D colormap in (**i**). A fixed hemodynamic delay value of 3.9 seconds is assumed and compensated for all pixels to derive the tuning phase from the raw response phase. **f.** Tonotopic maps derived by combining these two "temporally reversed" sessions (**d-e**) together. The hemodynamic delay of each pixel was individually estimated and canceled out in these maps. **g.** Hemodynamic delay maps derived by combining these two "temporally reversed" sessions (**d-e**) together. Each hemodynamic delay map only includes the top 10% of most responsive pixels. **h.** Histograms of hemodynamic delay values of the pixels in (**g**). Background rectangular gray shade: the acceptable delay range. Bottom number label: averaged delay within this range in each subject. The overall mean value of all subjects is 3.9 seconds. **i**. The 2D colormap for the response maps shown in (**d-f**). The upper display limit of response amplitude "M" is listed at the top right of each tonotopic map (**d-f**).

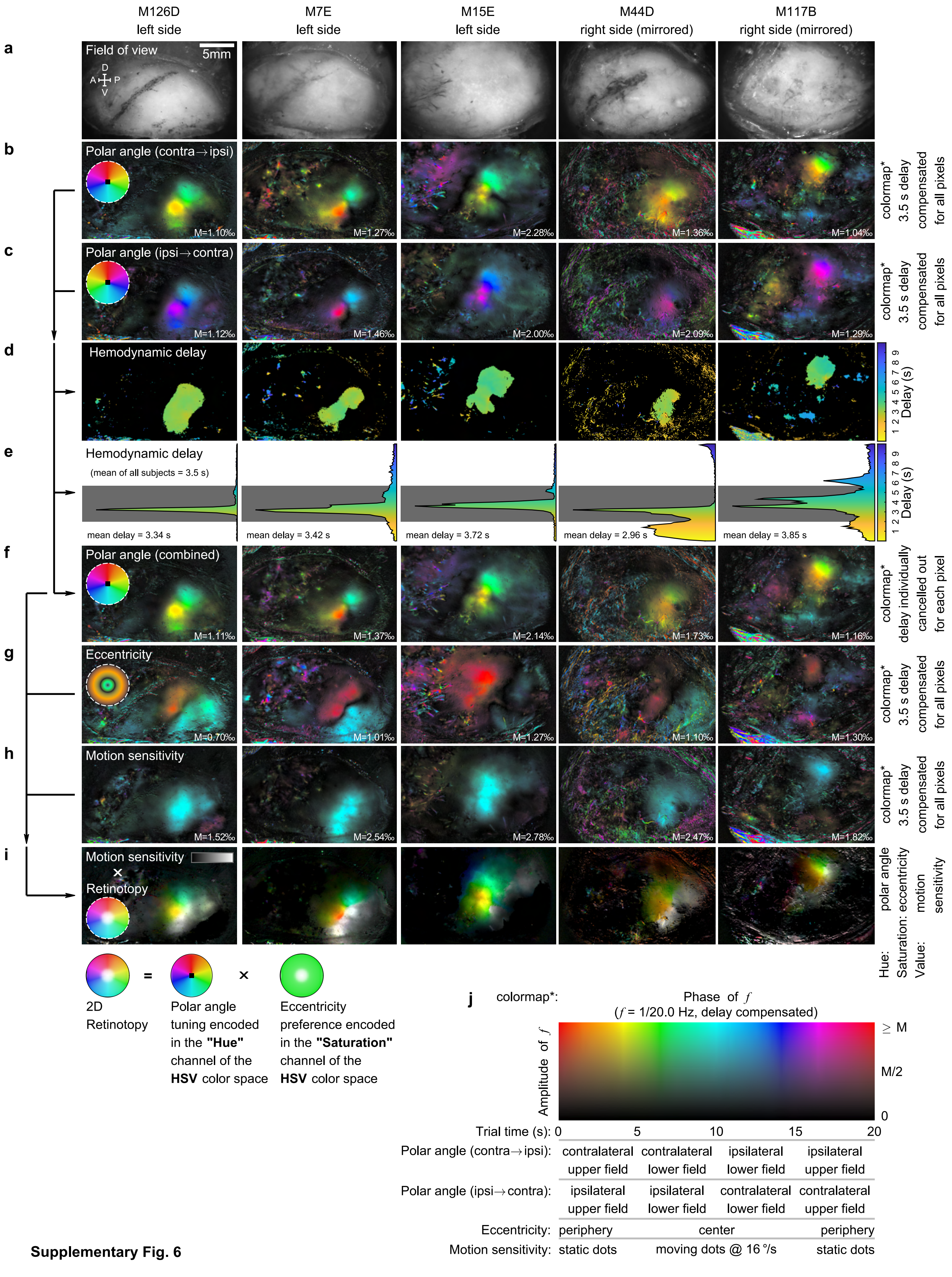

Supplementary Fig. 6

Supplementary Fig. 6. Retinotopy and motion-sensitivity mapping in all tested subjects

a. The field of view in each subject tested with the Fig. 5 experiments. Column title: subject's ID and imaged hemisphere side. A: anterior; P: posterior; D: dorsal; V: ventral. **b-c**. Retinotopic polar angle maps derived from the session with the motion range sweeping in a sequence across the contralateral-upper, contralateral-lower, ipsilateral-lower, and ipsilateral-upper visual fields relative to the imaging side (**b**) or the reverse (**c**). The tuning phase and amplitude are visualized separately as color and intensity in these maps (the same for **f-h**), according to the 2D colormap in (**j**). The colormap is transformed into a visual field color code and shown as a color wheel at the upper left (coded for left hemispheres). The upper display limit of response amplitude "M" is labeled at the bottom right of each map (the same for **f-h**). **d**, **f**. Hemodynamic delay (**d**) and retinotopic polar angle (**f**) maps derived by combining these two "temporally reversed" sessions (**b-c**) together. **e**. Histograms of hemodynamic delay values of the pixels in (**d**). Background rectangular gray shade: the acceptable delay range. **g**. Retinotopic eccentricity maps. A visual field color code for retinotopic eccentricity is transformed from the colormap in (**j**) and shown as a color wheel at the upper left. **h**. Motion-sensitivity maps. **i**. The summary map in each subject by combining the maps in (**f-h**) and encoding them into separate channels in the HSV color space. The retinotopic polar angle tuning (**f**) is encoded in the hue (color) channel, whereas the retinotopic eccentricity tuning (**g**) is encoded in the saturation (chroma) channel. A visual field color wheel incorporating these two features is shown at the lower left (for left hemispheres). Additionally, the motion sensitivity (**h**) is encoded in the value (intensity) channel. Together, it is evident that the motion sensitivity to moving dots extends across two retinotopically organized regions, each representing the contralateral visual field, with a polar angle reversal between them. **j**. The 2D colormap for the maps in (**b-c** and **f-h**).

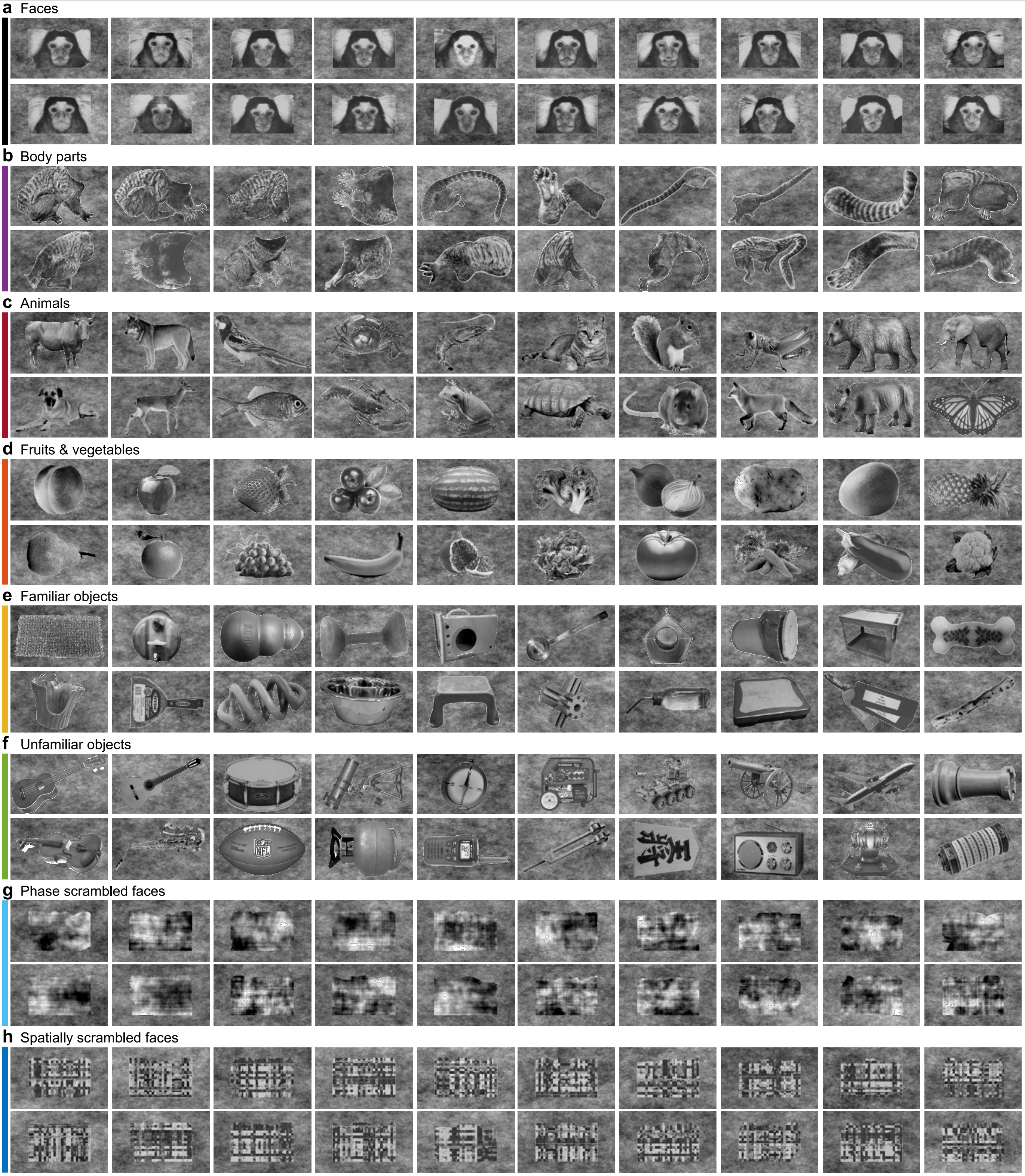

Supplementary Fig. 7. Face patch mapping stimuli

a-h. Eight visual form categories were used in the face patch mapping experiment, including faces (**a**), body parts (**b**), animals (**c**), fruits and vegetables (**d**), familiar objects (**e**), unfamiliar objects (**f**), phase scrambled faces (**g**), and spatially scrambled faces (**h**). Each category had 20 exemplars. Together, 160 exemplars used are all shown. The marmoset faces were used in the study by Hung et. al., 2015[52] and provided to us as a courtesy by the authors of that study.

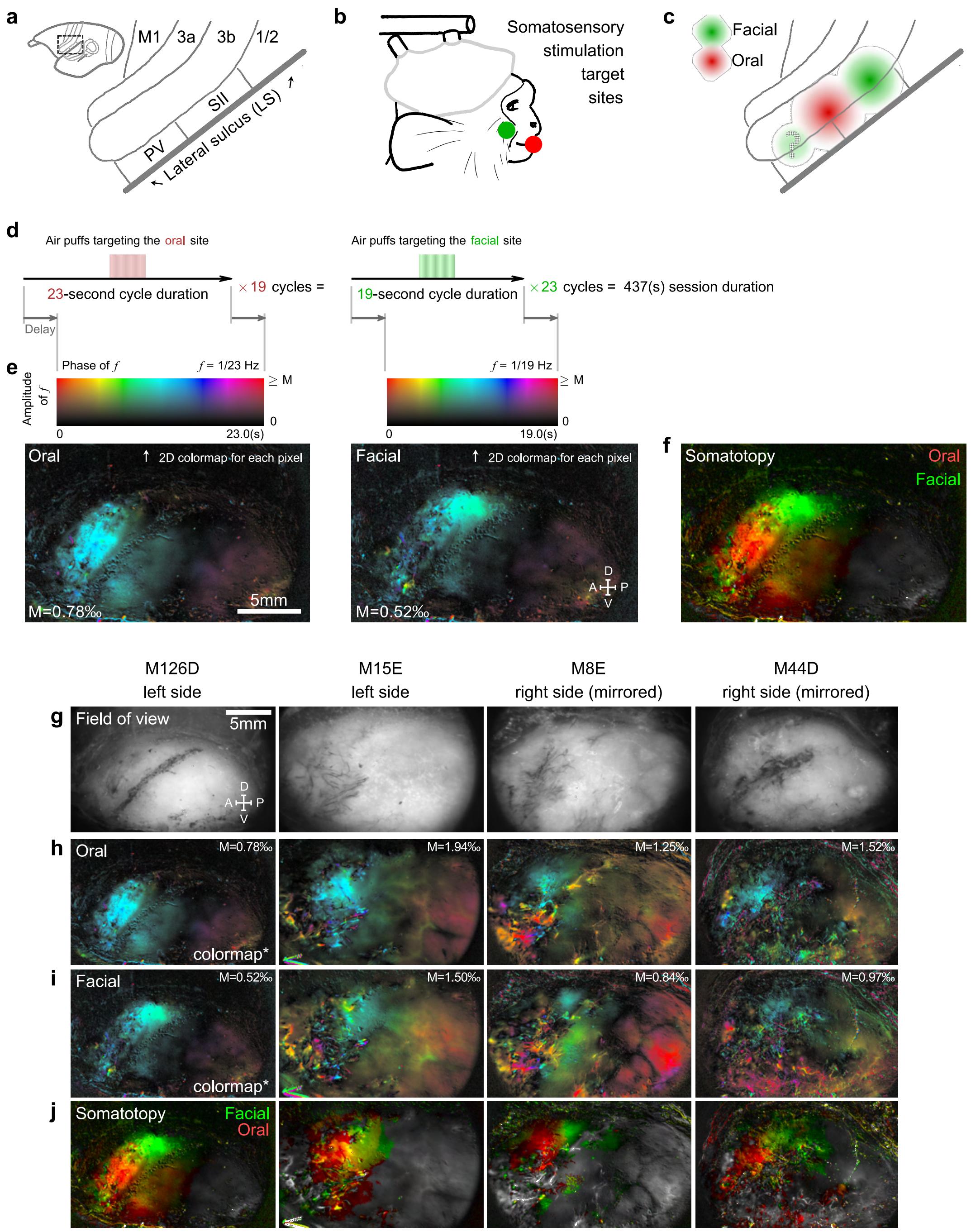

Supplementary Fig. 8. Through-skull somatotopy mapping

a. A sketch of marmoset somatosensory cortex[11] Noticeably, part of the secondary somatosensory area (SII) and parietal ventral area (PV) are also exposed outside the lateral sulcus. Electrophysiology mapping in anesthetized marmosets suggested that area 3b shares an orofacial representing border with both SII and PV[11], consistent with the orofacial region located in the experiment shown in Fig. 2e and Supplementary Fig. 4b. **b.** The target sites for air-puff stimulation. Red: oral cavity; green: contralateral cheek. The same colors also apply in (**c**, **d**, **f**, and **j**). **c.** An illustration of the oral and facial responsive regions in somatosensory cortex based on the results in (**e**-**j**). **d.** Stimulus design for testing whether the representations of the mouth and the face are topographically differentiable in marmoset somatosensory cortex. **e.** Response maps evoked by the oral (left) or facial (right) stimuli in subject M126D. For each pixel, the response phase and amplitude are visualized separately as color and intensity in these maps. Both types of stimuli evoked cortical activations in regions immediately anterodorsal to the putative lateral sulcus. **f.** A summary map of the orofacial somatotopic gradient. Oral (red) and facial (green) representations are well differentiable. The oral region appeared more anteroventral to the major facial region. **g.** The field of view in each tested subject. Column title: subject's ID and imaged hemisphere side. A: anterior; P: posterior; D: dorsal; V: ventral. **h**-**i**. The response maps evoked by the oral (**h**) or the facial (**i**) stimuli in each subject. All response maps are visualized in the same way as in (**e**). **j.** A summary map of the orofacial somatotopic gradient in each subject. Every subject showed an orofacial somatotopic gradient with the oral component at the more anteroventral side, and the major facial component at the more posterodorsal side, with minimal overlap between them.

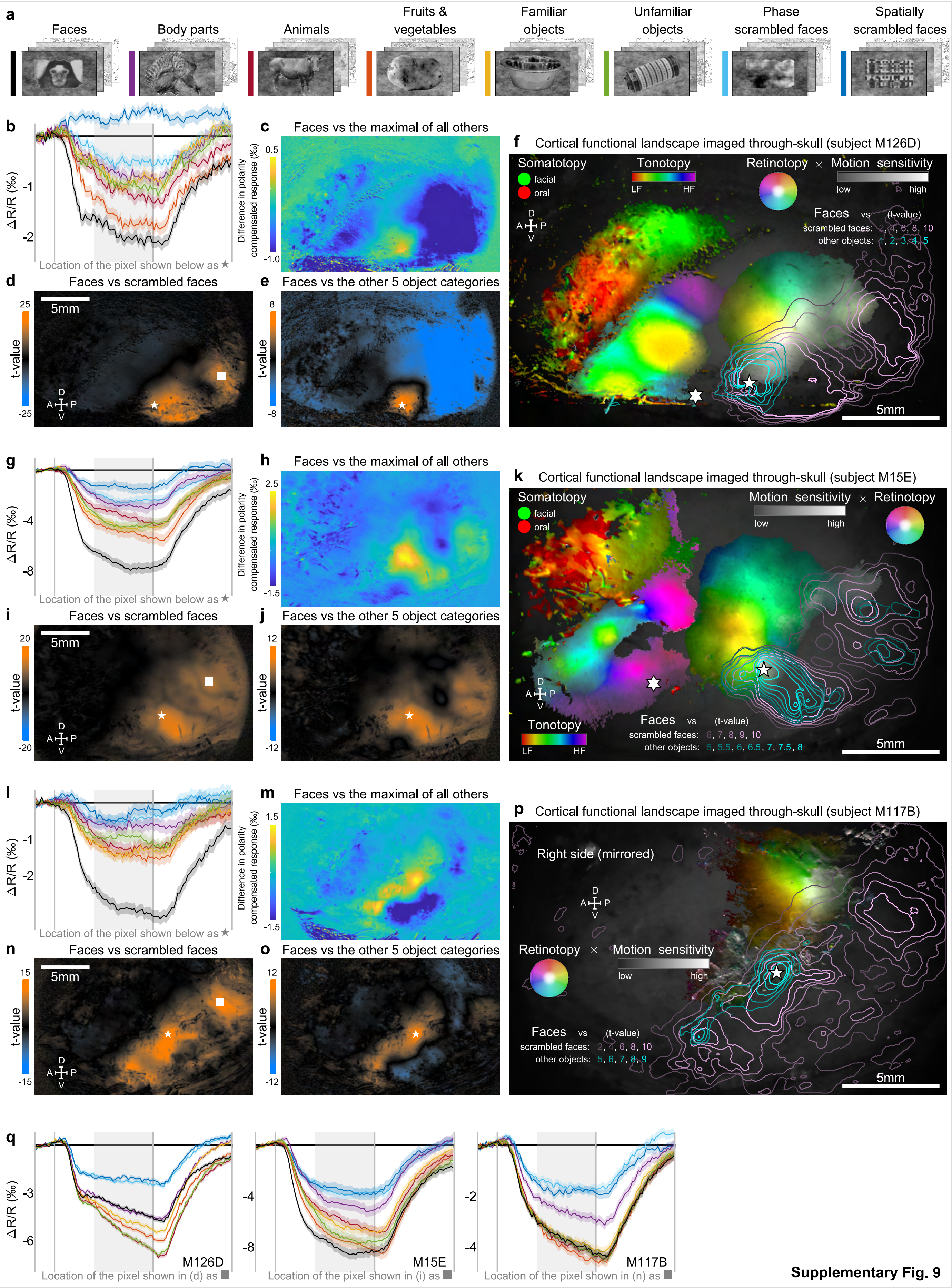

Supplementary Fig. 9

Supplementary Fig. 9. Face patch mapping in all tested subjects and their cortical functional landscapes.
a. Eight stimulus categories for face patch mapping (see also Fig. 6, Supplementary Fig. 7). The color codes for categories are also used in (**b**, **g**, **l**, and **q**). Mapping was performed in subjects M126D (**b-f**), M15E (**g-k**), and M117B (**l-p**). Maps acquired in subject M117B are mirrored. **b**, **g**, **l**. Response to each category of a face-sensitive pixel in the subject. The location of the pixel is labeled by a pentagram in the t-value maps below. The colors indicate categories as in (**a**). Solid curves: average response traces; shades: corresponding SEMs (n=218, 134, 234 trials for subjects M126D, M15E, M117B, respectively). The responses to the faces are the strongest among all categories, followed by the responses to other object categories and then the scrambled faces. The rectangular background gray shade indicates an averaging time window for calculating a response value of each trial. **c**, **h**, **m**. Differential response map between the mean response to the face category and the maximum of mean responses to all other categories (n=218, 134, 234 trials for each subject respectively). A positive value in the map indicates the pixel responded more strongly to faces than to any of the other seven stimulus categories. **d**, **i**, **n**. The t-value map for comparing faces versus scrambled faces. A t-value is calculated for each pixel by comparing its single-trial responses between those to the faces (n=218, 134, 234 trials) and those to the scrambled faces (n=436, 268, 468 trials for each subject respectively). Preference for unscrambled face in the map (orange color: high t-values) implicates involvement in visual form processing[92]. **e**, **j**, **o**. The t-value map for comparing the face category (n=218, 134, 234 trials) versus all other five object categories (n=1090, 670, 1170 trials for each subject respectively). The face-over-object sensitivity in the map (orange color: high t-values) defines the location of a face patch. **f**, **k**, **p**. The cortical functional landscape summarized from modality-specific maps. The maps are overlaid on a dimmed image of the recording chamber in each subject. The motion-sensitive region is shown with retinotopic tunings together in the "HSV" color space (hue [color]: polar angle; saturation [chroma]: eccentricity; value [intensity]: motion sensitivity, see also Supplementary Fig. 6i). The t-value maps are shown by the iso-t-value contour lines. Pentagram: a face-sensitive pixel (the same location as in previous t-value maps). Hexagram: a pixel from the newly discovered tonotopic gradient (see also Fig. 3h) representing frequencies commonly found in marmoset vocalizations (~4-16kHz[53]). The face patch (outlined by the cyan contours) is largely overlapped with both the moving-dots-sensitive region and the retinotopic region representing the lower center of the visual field (see also Supplementary Fig. 10). **q**. Responses of another exemplar pixel in the posterior face-scrambling sensitive region. The pixel location is labeled by a square in (**d**, **i**, and **n**) for each subject. The same plotting scheme in (**b**, **g**, **l**) applies.

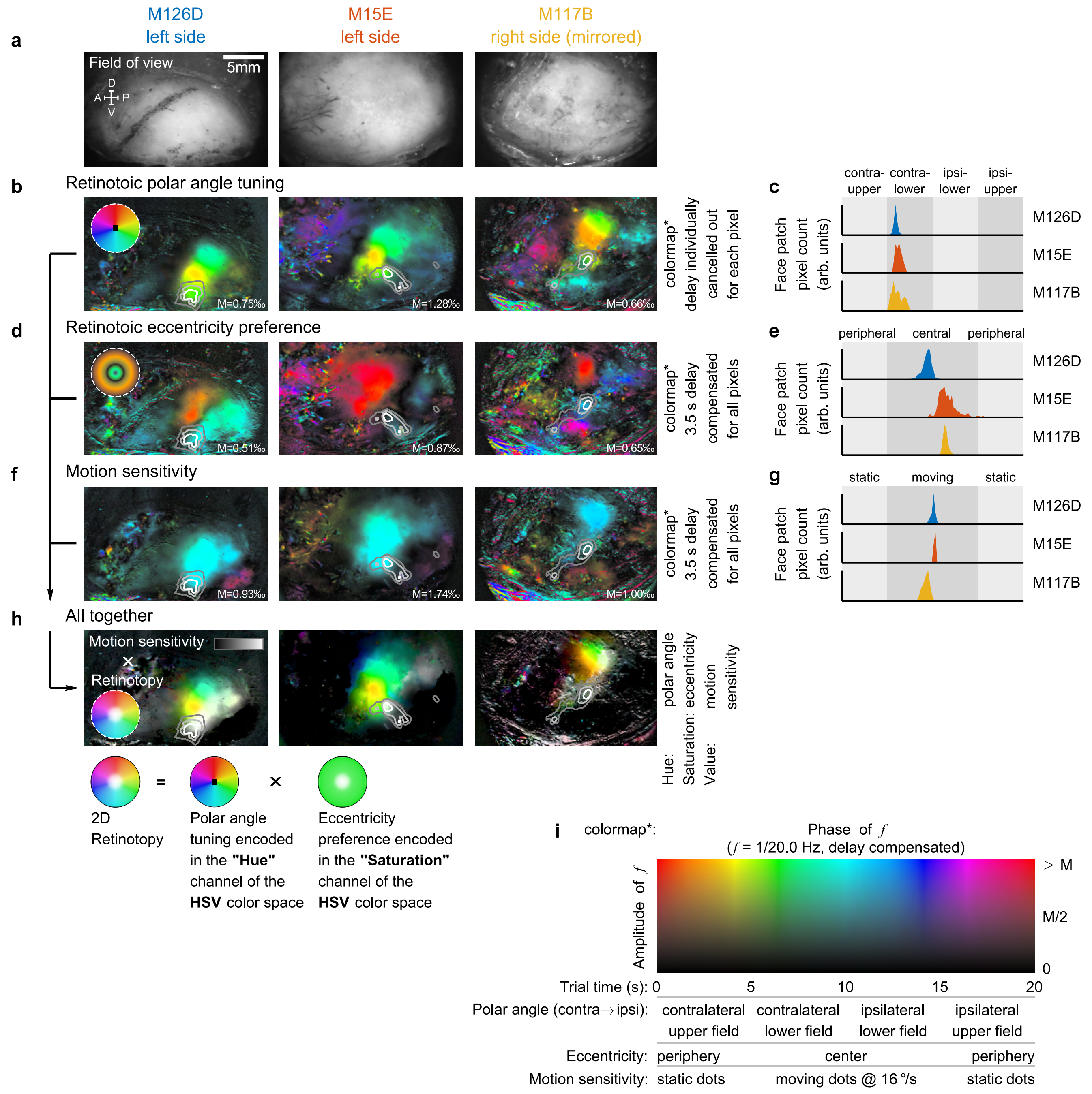

Supplementary Fig. 10. Registrations of the PD face patch to the retinotopic and motion sensitivity maps

a. The field of view in each subject mapped in the face patch experiment (Fig. 6). Column title: subject's ID and imaged hemisphere side. The color scheme is also followed in (**c**, **e**, and **g**). A: anterior; P: posterior; D: dorsal; V: ventral. **b**, **d**, **f**. Registrations of the PD face patch to other functional maps (Supplementary Fig. 6f, g, and h), including the retinotopic polar angle (**b**) and eccentricity (**d**) maps, and the motion sensitivity map (**f**). The tuning phase and amplitude are visualized separately as color and intensity in these map, according to the 2D colormap in (**i**). The colormap can be transformed into a visual field color code and shown as a color wheel at the upper left in the retinotopic maps (**b** and **d**). The upper display limit of response amplitude “M” is labeled at the bottom right of each map. The face patch is circled by iso-t-value contours as shown in Supplementary Fig. 9f, k, and p (the 1st, 3rd, and the 5th inner-most). **c**, **e**, **g**. Histograms of face patch pixels for their tunings under other functional contrasts. The face patch pixels were defined by the inner-most iso-t-value contour in (**b**, **d**, and **f**). In each of the three subjects, the polar-angle tuning of the face patch (**c**) is significantly below the horizontal meridian and sits within the quarter of the contralateral lower visual field (p-values = 1.46×10^{-139}, 1.60×10^{-62}, and 2.97×10^{-59}, Wilcoxon signed-rank test, single-sided, n=861, 369, and 349 pixels, respectively). The face patch also prefers the center over the periphery of the visual field (**e**), as well as the moving dots over the static dots (**g**), in each subject. **h**. Co-registration of the PD face patch to the summary map (Supplementary Fig. 6i) that combines all retinotopic and motion sensitivity maps. These maps are encoded into separate channels in the HSV color space. **i**. The 2D colormap for the response maps shown in (**b**, **d**, and **f**).

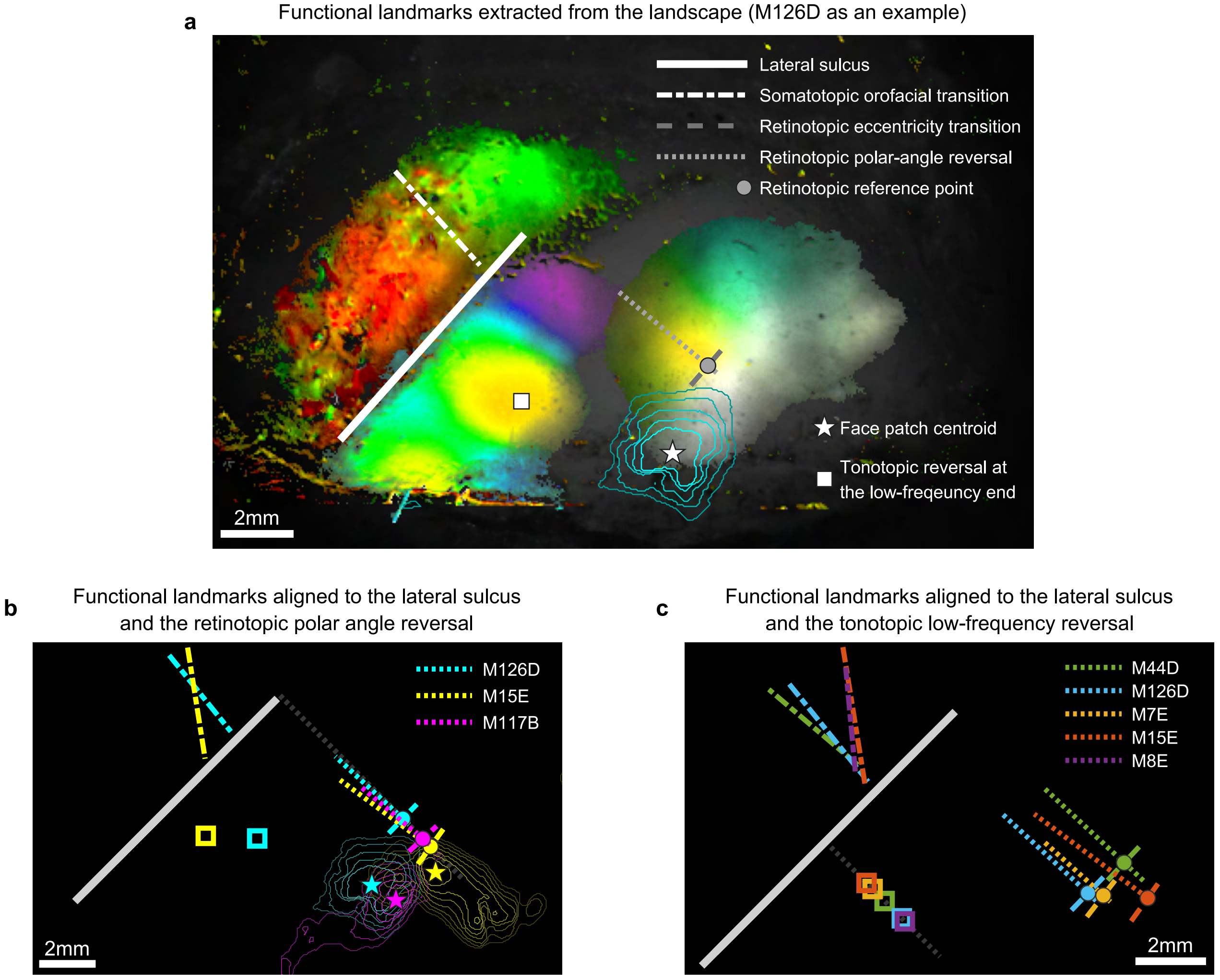

Supplementary Fig. 11. Quantification of individual variation in the functional landscape
a. An illustration for functional landmarks determined in each subject to be further co-registered together. A retinotopic reference point is defined as the crossing point between the marked retinotopic polar-angle reversal and the retinotopic eccentricity transition line. **b**. Three face patch tested subjects were co-registered together, by first aligning their lateral sulci together and then aligning their retinotopic reference points in a line perpendicular to the co-registered lateral sulcus. The subjects M126D and M15E had their centroids of the PD face patch separated by 2.3 mm. The cores of the face patch in these subjects (defined by the inner-most iso-t-value contour) were largely non-overlap with each other. These results demonstrate individual variation in the location of the PD face patch is at a scale that is comparable to the size of the face patch itself. **c**. Five tonotopy tested subjects were co-registered together, by first aligning their lateral sulci together and then aligning their low-frequency tonotopic reversals in a line perpendicular to the co-registered lateral sulcus. The distance of the low-frequency tonotopic reversal from the lateral sulcus varied between 1.6 mm to 3.2 mm among different subjects, suggesting a difficulty for localizing the functional reversal using the reference of the lateral sulcus. The retinotopic reference points also varied among subjects in a range of ~2 mm in the directions of both parallel and perpendicular to the lateral sulcus. These results further demonstrate the scale of individual variation in the functionally defined areas can be up to ~2 mm in marmosets, ~6% of their brain size.
