# Supplementary material for: Mesoscopic landscape of cortical functions revealed by through-skull wide-field optical imaging in marmoset monkeys
Source: Nat Commun. 2022 Apr 26;13:2238. doi: 10.1038/s41467-022-29864-7 (PMC9042927; doi:10.1038/s41467-022-29864-7)
Supplement: Supplementary file 4 — Reporting Summary [file 41467_2022_29864_MOESM4_ESM.pdf]

nature portfolio

Corresponding author(s): Xindong Song
Xiaoqin Wang

Last updated by author(s): Mar 22, 2022

Reporting Summary

Nature Portfolio wishes to improve the reproducibility of the work that we publish. This form provides structure for consistency and transparency in reporting. For further information on Nature Portfolio policies, see our Editorial Policies and the Editorial Policy Checklist.

Statistics

For all statistical analyses, confirm that the following items are present in the figure legend, table legend, main text, or Methods section.

n/a	Confirmed	
☐	☒	The exact sample size (*n*) for each experimental group/condition, given as a discrete number and unit of measurement
☐	☒	A statement on whether measurements were taken from distinct samples or whether the same sample was measured repeatedly
☐	☒	The statistical test(s) used AND whether they are one- or two-sided<br>*Only common tests should be described solely by name; describe more complex techniques in the Methods section.*
☒	☐	A description of all covariates tested
☒	☐	A description of any assumptions or corrections, such as tests of normality and adjustment for multiple comparisons
☐	☒	A full description of the statistical parameters including central tendency (e.g. means) or other basic estimates (e.g. regression coefficient) AND variation (e.g. standard deviation) or associated estimates of uncertainty (e.g. confidence intervals)
☐	☒	For null hypothesis testing, the test statistic (e.g. *F*, *t*, *r*) with confidence intervals, effect sizes, degrees of freedom and *P* value noted<br>*Give P values as exact values whenever suitable.*
☒	☐	For Bayesian analysis, information on the choice of priors and Markov chain Monte Carlo settings
☒	☐	For hierarchical and complex designs, identification of the appropriate level for tests and full reporting of outcomes
☐	☒	Estimates of effect sizes (e.g. Cohen's *d*, Pearson's *r*), indicating how they were calculated

Our web collection on statistics for biologists contains articles on many of the points above.

Software and code

Policy information about availability of computer code

Data collection	Optical imaging data collection routine was performed with MATLAB R2018a and custom MATLAB code. The code is openly accessible through https://github.com/x-song-x/XINTRINSIC (the commit on Dec, 2, 2019), and https://github.com/x-song-x/ChKshared (the commit on Dec, 7, 2019). Monte Carlo simulation was performed with MCXLAB v2019.4 (http://mcx.space) and MATLAB R2018a or higher. Structural MRI data collection was performed with ParaVision 6.0.1.
Data analysis	Optical imaging data analysis routine was performed with MATLAB R2018a or later and custom MATLAB code, openly accessible through https://github.com/x-song-x/fluffy-goggles (the commit on Aug 25, 2020).

For manuscripts utilizing custom algorithms or software that are central to the research but not yet described in published literature, software must be made available to editors and reviewers. We strongly encourage code deposition in a community repository (e.g. GitHub). See the Nature Portfolio guidelines for submitting code & software for further information.

Data

Policy information about availability of data

All manuscripts must include a data availability statement. This statement should provide the following information, where applicable:

- Accession codes, unique identifiers, or web links for publicly available datasets
- A description of any restrictions on data availability
- For clinical datasets or third party data, please ensure that the statement adheres to our policy

The raw data supporting the current study have not been deposited in a public repository because of the large size of the dataset but are available from the corresponding authors upon reasonable request.

Field-specific reporting

Please select the one below that is the best fit for your research. If you are not sure, read the appropriate sections before making your selection.

☒ Life sciences ☐ Behavioural & social sciences ☐ Ecological, evolutionary & environmental sciences

For a reference copy of the document with all sections, see nature.com/documents/nr-reporting-summary-flat.pdf

Life sciences study design

All studies must disclose on these points even when the disclosure is negative.

Sample size

Eleven marmosets were tested in this study. This is a large sample-size for primate neurophysiology research and was chosen to ensure that our results were robust across individual animals. The usage of animals in each experiment towards landscape mapping was described in detail in the Supplementary Table 1 (also with their sex and age). Specifically, the modality-parcellation, somatotopy-mapping, tonotopy-mapping, retinotopy-mapping, and face-patch mapping experiments were each conducted in 6, 4, 5, 5, and 3 animals, respectively. The wavelength-effect on tonotopy mapping experiment was conducted with 3 additional animals. Another 2 animals were further tested with the tonotopy mapping experiment using green light only and shown in Fig. 3i

All experiments, excepted for the face-patch mapping experiment, were designed based on a Fourier approach (Kalatsky et. al. 2003) to potentially improve the SNR and reduce the experimental time. In earlier pilot experiments, we found the results across repeated sessions on the same experiment with our current preparation were generally robust when a repetition number of ~20 was used. Thus, repetition numbers of 18, 19, 20, 22, and 23 were used in these experiments.

For the face-patch experiment, subjects M126D, M15E, and M117B each finished 218, 134, and 234 experimental cycles, respectively. These numbers are comparable to the sample-sizes in other marmoset functional neuroimaging studies using fMRI (e.g. Hung et. al. 2015).

Data exclusions

No data were excluded from the analyses for any available animal in each experiment (labeled "[Y]es" in Supplementary Table 1). For each animal that was not available in a certain experiment, the reason was labeled in Supplementary Table 1.

Replication

Each experiment in the subject M126D, shown in the main figures, was replicated in several other animals. The claims on the functional maps were largely confirmed in all tested subjects. The modality-parcellation experiment was replicated in totally 6 animals and the individual results were shown in Supplementary Fig. 4. The tonotopy-mapping experiment was replicated in totally 10 animals and the individual results were shown in Fig. 3i, Supplementary Fig. 2 and 5. The retinotopy-mapping experiment was replicated in totally 5 animals and the individual results were shown in Supplementary Fig. 6. The somatotopy-mapping experiment was replicated in totally 4 animals and the individual results were shown in Supplementary Fig. 8. The face-patch mapping experiment was replicated in totally 3 animals and the individual results were shown in Supplementary Fig. 9.

To validate the signal we imaged through-skull was indeed the intrinsic optical signal originated in the cortex. Three additional experiments were conducted.

The first experiment was to compare the map imaged through-skull to the map imaged through a subsequently implanted cranial window. A cranial window was chronically implanted over the putative auditory cortex in one of the subjects. The tonotopy-mapping experiment was replicated and performed through the cranial window. The through-window results were listed in parallel to the through-skull results in Fig. 4. The through-skull pattern was replicated as shown in the through-window pattern, suggesting the signal we measured through-skull was indeed the intrinsic optical signal in the cortex.

The second experiment was to test whether the through-skull signal, like the cortical intrinsic signal, shows a similar dependence on the measurement-wavelength. Six wavelengths were tested in the same subject (Supplementary Fig. 1). The results can be well explained by the wavelength-dependence in the absorption change following a cortical hemodynamic response (Hillman 2007, 2014), suggesting the through-skull signal exhibits key features as of the cortical intrinsic signal.

The third experiment was to test whether the intrinsic signal in the auditory cortex, like many previous studies have suggested, is not robust at red wavelengths. Six wavelengths were used to map tonotopy in three additional subjects (Supplementary Fig. 2). The results showed robust maps with blue or green lights, but not with the red, far-red, or amber lights, consistent with the previous intrinsic imaging in auditory cortex across a variety of species.

Randomization

A Fourier-based phase-encoding approach that requires specific temporal arrangement in the stimuli was utilized in the modality-parcellation, somatotopy-mapping, tonotopy-mapping, and retinotopy-mapping experiments. Temporal randomization is thus not applicable in these experiments.

The initial positions of the moving dots, as well as the moving directions of subsequently re-generated dots, were randomized in the modality-parcellation and the retinotopy-mapping experiments.

In the face-patch mapping experiment, several layers of randomization were performed through the stimulus generation process. (1) For each stimulus frame, the pink-noise background was generated with randomized phases among frequency components. (2) For each stimulus trial, the order of 20 exemplars of the category was randomized. (3) For each experimental cycle, the presentation order of the 8 category trials was randomized.

Random group allocation is not relevant since the current study has no experiment that requires subject grouping.

Blinding	It is not possible to blind our subjects to the sensory stimuli in any of our experimental sessions. The imaging analysis was performed with the same computational routine in which blinding is not relevant

Reporting for specific materials, systems and methods

We require information from authors about some types of materials, experimental systems and methods used in many studies. Here, indicate whether each material, system or method listed is relevant to your study. If you are not sure if a list item applies to your research, read the appropriate section before selecting a response.

Materials & experimental systems

n/a	Involved in the study
☒	☐ Antibodies
☒	☐ Eukaryotic cell lines
☒	☐ Palaeontology and archaeology
☐	☒ Animals and other organisms
☒	☐ Human research participants
☒	☐ Clinical data
☒	☐ Dual use research of concern

Methods

n/a	Involved in the study
☒	☐ ChIP-seq
☒	☐ Flow cytometry
☐	☒ MRI-based neuroimaging

Animals and other organisms

Policy information about studies involving animals; ARRIVE guidelines recommended for reporting animal research

Laboratory animals	The common marmoset (Callithrix jacchus) was used in the current study, including 4 female, 7 males. The animals ranged from 44 to 81 months old during data acquisition.
Wild animals	No wild animals were involved in the current study.
Field-collected samples	No field-collected samples were involved in the current study.
Ethics oversight	All experimental procedures conformed to local and US National Institutes of Health guidelines and were approved by the Johns Hopkins University Animal Use and Care Committee.

Note that full information on the approval of the study protocol must also be provided in the manuscript.

Magnetic resonance imaging

Experimental design

Design type	Structural scan
Design specifications	22 minutes scan
Behavioral performance measures	No behavioral tasks

Acquisition

Imaging type(s)	structural
Field strength	11.7 T
Sequence & imaging parameters	FLASH 3D Sequence, FOV: 30*30*35 mm, matrix size: 200*200*234, slice thickness: 0.15 mm, orientation: coronal, TE: 3.6ms, TR: 25 ms, flip angle: 20 degrees
Area of acquisition	whole brain

Diffusion MRI ☐ Used ☒ Not used

Preprocessing

Preprocessing software	Freesurfer V6
Normalization	N/A
Normalization template	N/A

Noise and artifact removal | N/A

Volume censoring | N/A

Statistical modeling & inference

Model type and settings | N/A

Effect(s) tested | N/A

Specify type of analysis: ☒ Whole brain ☐ ROI-based ☐ Both

Statistic type for inference (See Eklund et al. 2016) | N/A

Correction | N/A

Models & analysis

n/a	Involved in the study
☒	☐ Functional and/or effective connectivity
☒	☐ Graph analysis
☒	☐ Multivariate modeling or predictive analysis
